# Psychosocial function, legal involvement and violence in mental disorder

**DOI:** 10.1192/j.eurpsy.2021.2250

**Published:** 2021-12-03

**Authors:** Alec Buchanan, Kelly E. Moore, Brian Pittman, Sherry A. McKee

**Affiliations:** 1 Law and Psychiatry Division, Yale School of Medicine, New Haven, Connecticut 06519, USA; 2 VA Connecticut Health Care System, West Haven, Connecticut 06516, USA; 3 Department of Psychology, East Tennessee State University, Johnson City, Tennessee 37614, USA; 4 Department of Psychiatry, Yale School of Medicine, New Haven, Connecticut 06519, USA

**Keywords:** Diagnosis, violent behavior, psychosocial function, incarceration, arrest

## Abstract

**Background:**

The correlates of legally significant outcomes that have been identified in people with mental disorders are of limited value in understanding the mechanisms by which these outcomes occur.

**Aims:**

To describe the relationships between mental disorder, impaired psychosocial function, and three legally significant outcomes in a representative sample of the US population.

**Methods:**

We used a population survey, the National Epidemiologic Survey on Alcohol and Related Conditions (NESARC-III, sample size 36,309), to identify people who self-reported serious trouble with the police or the law over the past 12 months and two lifetime outcomes, being incarcerated and engaging in violence to others. DSM-5 categories were generated using the Alcohol Use Disorder and Associated Disabilities Interview Schedule-5. Psychosocial function was assessed using social and role-emotional function scores of the 12-Item Short-Form Health Survey Version 2.

**Results:**

Participants with mental disorder, but not people with no diagnosis, who reported serious trouble with the police or with the law during the previous 12 months reported significantly worse psychosocial function than those who did not report such trouble. The size of the statistical effect varied by diagnosis, moderate for some forms of mental illness and for alcohol abuse and nonsignificant for drug abuse and the personality disorders. Effect sizes were largest for diagnoses where legally significant outcomes were least common.

**Conclusions:**

The effect of impaired psychosocial function, for instance in disrupting family and social networks that would otherwise protect against these legally significant outcomes, warrants further investigation in studies with longitudinal designs.

## Introduction

The relationship between mental disorder and legally significant outcomes such as legal trouble, incarceration, and violence are clinically important but empirically unresolved. Bivariate analyses of population surveys and cohort samples most frequently focus on violence and report rates that are three to four times higher than in the general population for depression, anxiety disorder, and eating disorder [1] and for categories that combine diagnoses such as major mental disorder [2]. Rates are of violence are 10 times higher for substance use disorder [3] and two to three times higher again for people with multiple psychiatric diagnoses [4]. Sociodemographic characteristics and past behavior are also known correlates of legally significant outcomes and the statistical effects of all forms of mental disorder are smaller when multivariate approaches are used to control for these variables [5,6].

Only in psychosis, however, have these findings been followed by significant progress in identifying mechanisms that may explain the association between mental disorder, on the one hand, and legal trouble, incarceration and violence, on the other [7]. In particular, the relative contributions of symptoms and signs of illness, of labeling and stigma and of known risk factors that are unrelated to mental state, remain largely unknown. Given the large number of diagnoses involved, one way forward is to identify signs and symptoms of mental disorder that can be used across diagnoses to identify the times and circumstances in which legal involvement is most likely, to suggest explanations and, hence, to inform interventions.

One candidate for signs and symptoms that may offer clues as to mechanism is the impairment of psychosocial function that all mental disorders cause [8]. Functional impairment is a recognized marker of illness severity and a criterion for diagnosis in each of the recognized international classifications of mental disorder [9,10]. However, the relationship between diagnosis, function and legal involvement has been studied only infrequently and in treatment samples, where selection bias limits the generalizability of findings [11,12]. The limited data that do exist suggest that for some diagnoses function exerts an interactive effect, protecting against violent behavior in people with psychosis who have frequent social contacts [13], or a “curvilinear” effect, whereby violent behavior is less frequent at extremes of social function and more frequent when function is moderate [14].

We used the third wave of a national community survey sample, the National Epidemiologic Survey on Alcohol and Related Conditions (NESARC-III), to test whether people with mental disorders who describe serious trouble with the law or the police, being incarcerated or engaging in violence also describe impaired psychosocial function.

## Method

### Sample and procedures

The NESARC-III is a cross sectional representative survey of the civilian US population sponsored by the National Institute on Alcohol Abuse and Alcoholism (NIAAA). The survey conducted in-person interviews with noninstitutionalized US adults, including the residents of group and rest homes, between April of 2012 and June of 2013. The final sample size was 36,309. The person-level response rate was 84%. Consent procedures were approved by the National Institutes of Health. Detailed descriptions of the methodology have been published [15,16].

### Variables

Diagnoses for mood disorders, anxiety disorders, PTSD, eating disorders, substance use disorders, and personality disorders were generated using the Alcohol Use Disorder and Associated Disabilities Interview Schedule (AUDADIS)-5, a structured interview generating DSM categories. Test–retest reliability for the AUDADIS-5 ranges from fair to excellent and is similar to that for other structured diagnostic interviews [17]. Personality disorder diagnoses were generated for a participant’s lifetime. Other diagnoses were generated for two time periods, past year and lifetime. We used the response on two NESARC-III items, “In the last 12 months, did a doctor or other health professional tell you that you had schizophrenia or a psychotic illness or episode?” and, “Did this happen before 12 months ago?” to generate two further variables, past year and lifetime “schizophrenia/psychosis.”

We created three binary outcome variables relating to behavioral outcomes. One used the NESARC-III item, “Please tell me if you have had any of the following experiences in the last 12 months … did you have serious trouble with the police or the law?” The second measured lifetime incarceration using the NESARC-III item, “Since you were 18, were you ever in jail, prison or a correctional facility?” The third measured a lifetime history of violence using, as elsewhere [18], a positive response to any of 7 NESARC-III items relating to violent behavior occurring since the age of 15: stole directly (by mugging or making threats with a weapon); forced sexual activity; got into fights one started; physically hurt someone; exchanged blows in a fight; used a weapon; and hit someone causing injury or hospitalization.

We measured psychosocial function using the 12-Item Short-Form Health Survey Version 2 (SF-12v2) [19] which generates norm-based disability scores with a mean (SD) of 50 (10) and a range of 0–100. Lower scores indicate greater disability. Social function is defined by the SF-12v2 as the extent to which a subject perceives mental health problems as having interfered with their normal social activities [20,21]. One item measuring social functioning asks, “During the past 4 weeks, how much of the time have your physical health or emotional problems interfered with your social activities like visiting with friends, relatives, and so forth.”

Role-emotional functioning in the SF-12v2 addresses the extent to which a subject’s symptoms lead them to cut down the amount of time spent on work, to accomplish less than they would like at work and to carry out daily activities less carefully than usual. Items include, “During the past 4 weeks, tell me how much of the time you have had any of the following problems with your work or other regular daily activities as the result of any emotional problems, such as feeling depressed or anxious,” “How much of the time have you accomplished less than you would like?” and, “How much of the time have you not done work or other activities as carefully as usual?”

Testing shows the SF-12v2 to be a reliable and valid measure of function [19]. We did not include two scores that it can be used to generate, mental health and the mental component summary, because of the similarity of some of their items (feeling “nervous” or “downhearted and blue”) to AUDADIS-5 diagnostic criteria. We included only those personality disorder diagnoses for which AUDADIS-5 reliability data have been published: borderline, antisocial and schizotypal [17].

### Statistical analyses

We analyzed data on all subjects from NESARC-III for whom data on level of function were available (*n* = 36,293) using the logistic and linear regression survey procedures in SAS, version 9.4 [22]. These procedures allowed us to incorporate the stratification, clustering, and unequal weighting of the study sampling design and enabled the results to reflect three sociodemographic characteristics (age, sex, and self-defined ethnicity) of the US population.

We calculated rates for each outcome for each diagnosis. We used 12-month diagnoses to calculate rates for the 12-month outcome (serious trouble with the police or the law) and lifetime diagnoses to calculate rates for lifetime outcomes (incarceration and violence others). Rates were compared using logistic regression to generate odds ratios (ORs). We calculated mean social functioning and role-emotional functioning scores for subjects who did and who did not meet criteria for each outcome variable. The differences between these means were compared using linear regression. We report statistical significance and effect size. We describe effect size using Cohen’s *d.* We refer to effect sizes of between 0.2 and 0.5 as “small” and those of 0.5 and over as “moderate.”

## Results

The sample is described in Table 1. A total of 8,532 participants (23.5%) received a diagnosis of mental illness for the previous 12 months. Of these, 3.4% had encountered serious trouble with the police or the law compared with only 0.7% of participants with no 12-month diagnosis (OR of 5.6; 95% CI 4.6–6.9). The equivalent ORs for any substance abuse diagnosis and any personality disorder were 10.5 (95% CI 8.6–12.7) and 10.6 (95% CI 8.3–13.5), respectively. Among individual diagnoses, ORs were highest for drug abuse (OR 19.9; 95% CI 15.5–25.5), schizophrenia/psychosis (17.5; 95% CI 10.6–28.9) and antisocial personality disorder (16.6; 95% CI 12.2–22.6) and lowest for eating disorders (2.3; 95% CI 1.0–5.6) and specific phobias (4.3; 95% CI 3.0–6.2).

**Table 1.** Description of sample: diagnosis and outcomes variables (*n* = 36,293).

Diagnosis			Serious legal trouble past 12 months		Lifetime incarceration		Lifetime violence to others	
	*n* 12 months	*n* life	*n* (%)	OR (95% CI)^a^	*n* (%)	OR (95% CI)^a^	*n* (%)	OR (95% CI)^a^
No diagnosis	22618	17775	254 (0.7%)	Ref	809 (4.6%)	Ref	1660 (9.4%)	Ref
Any SU, MI, or PD	13675	18518	502 (3.7%)	6.1*** (5.1; 7.4)	3321 (18.0%)	4.7*** (4.2; 5.2)	6547 (35.4%)	4.9*** (4.5; 5.3)
Any SU or MI	12126	17755	467 (3.9%)	6.4*** (5.3; 7.7)	3181 (18%)	4.7*** (4.2; 5.2)	6117 (34.5%)	4.7*** (4.3; 5.1)
Any MI or PD	10797	13843	394 (3.6%)	6.2*** (5.2; 7.5)	2402 (17.4%)	4.5*** (4.0; 5.1)	5419 (39.2%)	5.8*** (5.3; 6.2)
Any SU or PD	9514	13219	462 (4.9%)	8.3*** (6.9; 10.0)	3034 (23.1%)	6.3*** (5.6; 7.1)	5681 (43.1%)	6.6*** (6.1; 7.2)
Comorbid SU and MI	2214	5470	171 (7.7%)	14.0*** (11.3; 17.4)	1428 (26.2%)	7.4*** (6.6; 8.4)	2743 (50.2%)	8.8*** (8.1; 9.7)
Comorbid SU and PD	2039	3455	205 (10.1%)	19.5*** (15.7; 24.2)	1287 (37.4%)	13.2*** (11.5; 15.2)	2522 (73.0%)	24.3*** (21.5; 27.5)
Comorbid MI and PD	3480	4198	207 (5.9%)	10.6*** (8.5; 13.3)	1186 (28.3%)	8.9*** (7.8; 10.3)	1665 (20.6%)	17.3*** (15.4; 19.4)
Comorbid SU and PD and MI	1323	2671	132 (10.0%)	19.0*** (14.6; 24.6)	963 (36.1%)	12.4*** (10.7; 14.5)	1944 (72.8%)	23.7*** (20.8; 26.9)
Any MI	8532	12296	286 (3.4%)	5.6*** (4.6; 6.9)	1938 (15.8%)	4.0*** (3.6; 4.5)	4411 (35.9%)	5.0*** (4.6; 5.4)
Schizophrenia/Psychosis	337	902	28 (8.3%)	17.5*** (10.6; 28.9)	218 (24.2%)	6.5*** (4.9; 8.6)	381 (42.2%)	6.9*** (5.3; 9.0)
Any mood disorder	4894	8639	198 (4.0%)	7.1*** (5.7; 8.9)	1382 (16.1%)	4.1*** (3.6; 4.6)	3252 (37.7%)	5.4*** (4.9; 5.8)
Major depression	3961	7430	149 (3.8%)	6.6*** (5.1; 8.4)	1061 (14.4%)	3.5*** (3.1; 4.0)	2584 (34.8%)	4.7*** (4.3; 5.2)
Persistent depressive	1185	2017	52 (4.4%)	7.6*** (5.4; 10.8)	371 (18.5%)	5.0*** (4.2; 5.9)	849 (42.3%)	6.7*** (5.8; 7.7)
Bipolar 1	565	752	37 (6.5%)	12.7*** (8.0; 20.1)	235 (31.5%)	9.8*** (7.6; 12.5)	491 (65.5%)	17.0*** (14.0; 20.7)
Any anxiety disorder	4700	5989	155 (3.3%)	5.5*** (4.3; 7.0)	1108 (16.9%)	4.3*** (3.8; 4.9)	2313 (38.7%)	5.6*** (5.1; 6.2)
Specific phobia	2035	2279	51 (2.5%)	4.3*** (3.0; 6.2)	353 (15.5%)	3.9*** (3.2; 4.6)	853 (37.5%)	5.4*** (4.7; 6.2)
Social anxiety	980	1255	37 (3.8%)	5.9*** (3.9; 8.9)	274 (22.0%)	5.9*** (4.9; 7.2)	552 (44.1%)	7.0*** (6.0; 8.3)
Panic	1103	1811	52 (4.7%)	7.0*** (4.7; 10.3)	353 (19.6%)	5.2*** (4.2; 6.3)	797 (44.1%)	6.9*** (6.1; 7.9)
Agoraphobia	549	690	22 (4.0%)	6.1*** (3.5; 10.6)	158 (23.1%)	6.3*** (4.8; 8.3)	342 (49.7%)	8.4*** (7.0; 10.1)
Generalized anxiety	1908	2708	82 (4.3%)	7.2*** (5.2; 10.0)	505 (18.7%)	4.8*** (4.1; 5.6)	1170 (43.2%)	6.7*** (6.0; 7.6)
Posttraumatic stress	1778	2337	87 (4.9%)	7.7*** (5.6; 10.6)	531 (22.8%)	6.3*** (5.4; 7.4)	1248 (53.4%)	9.8*** (8.7; 11.1)
Eating disorder^b^	385	615	6 (1.6%)	2.3 (1.0; 5.6)	89 (14.5%)	3.8*** (2.7; 5.2)	251 (40.8%)	6.2*** (5.1; 7.5)
Any substance use disorder	5808	10929	352 (6.1%)	10.5*** (8.6; 12.7)	2671 (24.6%)	6.7*** (6.0; 7.6)	4449 (40.8%)	6.1*** (5.5; 6.7)
Alcohol abuse	5133	10000	306 (6.0%)	10.2*** (8.3; 12.5)	2388 (24.0%)	6.6*** (5.8; 7.4)	4032 (40.4%)	6.0*** (5.4; 6.6)
Drug abuse	1487	3548	154 (10.4%)	19.9*** (15.5; 25.5)	1342 (38.1%)	13.1*** (11.3; 15.1)	1960 (55.4%)	11.2*** (9.9; 12.5)
Any personality disorder^c^	NA	5745	315 (5.5%)	10.6*** (8.3; 13.5)	1650 (28.8%)	9.1*** (8.0; 10.4)	3754 (65.3%)	17.3*** (15.6; 19.1)
Borderline^c^	NA	4300	250 (5.8%)	11.3*** (8.9; 14.3)	1240 (28.9%)	9.4*** (8.2; 10.7)	2911 (67.7%)	19.4*** (17.4; 21.7)
Antisocial^c^	NA	1600	132 (8.3%)	16.6*** (12.2; 22.6)	714 (44.8%)	18.5*** (15.7; 21.8)	1356 (84.8%)	49.8*** (41.8; 59.4)
Schizotypal^c^	NA	2438	148 (6.1%)	12.7*** (9.7; 16.6)	685 (28.2%)	8.8*** (7.4; 10.5)	1522 (62.4%)	15.0*** (13.1; 17.1)

*Note: Sample is limited to people with data on functional impairment. Psychiatric categories are not mutually exclusive. Lower scores indicate poorer perceived functioning.*

*** *p* < 0.001.

a Data weighted to adjust for nonresponse.

b Includes bulimia and anorexia nervosa.

c Only lifetime personality disorder diagnoses are available.

A total of 12,296 participants (33.9%) received a lifetime mental illness diagnosis. Of these, 15.8% had been incarcerated and 35.9% had committed one or more act of violence. Compared to people with no diagnosis, legal involvement was again commoner in people with mental illness (lifetime incarceration OR 4.0 [95% CI 4.6–6.9]; lifetime violence OR 5.0 [95% CI 4.6–6.9]), substance abuse (lifetime incarceration OR 6.7 (95% CI 6.0–7.6); lifetime violence OR 6.1 (95% CI 5.5–6.7); and personality disorders (lifetime incarceration OR 9.1 [95% CI 8.0–01.4]; lifetime violence OR 17.3 [95% CI 15.6–19.1]). For both 12-month and lifetime measures, having comorbid diagnoses from multiple categories (mental illness, substance abuse, and personality) was associated with a higher OR for all three outcomes.

The association, broken down by diagnosis, between having encountered serious trouble with the police or the law over the past 12 months and impaired psychosocial function is shown in Table 2. Participants with a mental disorder who reported such trouble described lower levels of function than those who did not report such trouble. For participants without a mental disorder there was no effect. For specific phobia (0.57), social anxiety (0.52), agoraphobia (0.52), and eating disorder (0.60) effect sizes were moderate. For other forms of mental illness effect sizes were small (0.32–0.49). For alcohol abuse the effect size of social functioning was again small (0.34) and for drug abuse and personality disorders it was not significant. Role-emotional function showed a similar pattern to social function but with smaller effect sizes.

**Table 2.** Serious legal trouble (SLT) past 12 months: effect size of functional impairment by 12-month diagnosis.

Diagnosis past 12 months	Social functioning				Role-emotional functioning			
	SLT present Mean (SE)	SLT absent Mean (SE)	*p*	*d*	SLT present Mean (SE)	SLT absent Mean (SE)	*p*	*d*
No diagnosis	52.4 (0.78)	52.7 (0.09)	ns	0.03	49.5 (0.96)	50.4 (0.15)	ns	0.09
Any SU, MI, or PD	44.3 (0.80)	47.3 (0.14)	<0.001	0.25	42.9 (0.70)	45.2 (0.14)	<0.01	0.20
Any SU or MI	43.9 (0.84)	47.1 (0.16)	<0.001	0.26	42.6 (0.74)	45.0 (0.15)	<0.01	0.21
Any MI or PD	42.7 (0.94)	46.0 (0.17)	<0.01	0.26	41.7 (0.85)	44.1 (0.15)	<0.01	0.20
Any SU or PD	44.2 (0.84)	47.1 (0.17)	<0.01	0.24	42.9 (0.73)	45.1 (0.16)	<0.01	0.19
Comorbid SU and MI	40.0 (1.34)	43.7 (0.33)	<0.01	0.29	39.2 (1.25)	41.9 (0.29)	<0.05	0.23
Comorbid SU and PD	43.1 (1.30)	44.2 (0.34)	ns	0.09	41.1 (1.19)	42.6 (0.31)	ns	0.13
Comorbid MI and PD	38.7 (1.34)	41.6 (0.26)	<0.05	0.21	38.3 (1.19)	40.2 (0.25)	ns	0.16
Comorbid SU and PD and MI	38.2 (1.63)	41.5 (0.45)	ns	0.17	37.9 (1.53)	40.2 (0.42)	ns	0.19
Any MI	40.0 (1.03)	45.2 (0.19)	<0.0001	0.40	39.7 (0.94)	43.3 (0.17)	<0.001	0.30
Schizophrenia/Psychosis	31.3 (3.45)	36.8 (0.99)	ns	0.39	31.1 (2.79)	36.7 (0.89	ns	0.43
Any mood disorder	38.7 (1.24)	43.0 (0.28)	<0.001	0.32	38.2 (1.21)	41.3 (0.24)	<0.05	0.25
Major depression	39.1 (1.28)	43.3 (0.28)	<0.01	0.32	39.0 (1.10)	41.7 (0.25)	<0.05	0.22
Persistent depressive	33.7 (1.82)	39.7 (0.61)	<0.01	0.42	33.5 (1.78)	38.4 (0.53)	<0.05	0.37
Bipolar 1	39.9 (3.90)	40.8 (0.75)	ns	0.07	36.2 (4.34)	38.8 (0.65)	ns	0.21
Any anxiety disorder	37.5 (1.24)	44.6 (0.25)	<0.0001	0.54	37.4 (1.22)	43.0 (0.23)	<0.0001	0.45
Specific phobia	38.6 (2.47)	45.9 (0.36)	<0.01	0.57	38.9 (1.89)	44.5 (0.30)	<0.01	0.47
Social anxiety	34.5 (2.59)	41.7 (0.53)	<0.01	0.52	35.3 (2.73)	41.3 (0.48)	<0.05	0.47
Panic	34.4 (2.29)	39.7 (0.57)	<0.05	0.36	32.5 (2.13)	39.1 (0.53)	<0.01	0.50
Agoraphobia	32.1 (3.04)	39.4 (0.68)	<0.05	0.52	34.4 (2.19)	38.0 (0.68)	ns	0.28
Generalized anxiety	35.7 (1.64)	42.4 (0.40)	<0.001	0.49	33.8 (1.80)	40.8 (0.35)	<0.001	0.56
Posttraumatic stress	34.9 (1.81)	41.6 (0.42)	<0.001	0.48	35.6 (1.85)	40.1 (0.35)	<0.05	0.34
Eating disorder^a^	36.4 (3.78)	44.1 (0.75)	<0.05	0.60	34.6 (3.50)	43.4 (0.79)	<0.05	0.70
Any substance use disorder	45.1 (0.92)	48.7 (0.19)	<0.001	0.32	43.3 (0.84)	46.4 (0.19)	<0.001	0.29
Alcohol abuse	45.6 (0.99)	49.3 (0.17)	<0.001	0.34	44.0 (0.79)	47.0 (0.19)	<0.001	0.28
Drug abuse	43.9 (1.27)	44.6 (0.49)	ns	0.05	40.8 (1.24)	42.4 (0.47)	ns	0.13
Any personality disorder	42.5 (1.12)	44.6 (0.21)	ns	0.16	41.3 (1.00)	43.0 (0.20)	ns	0.14
Borderline	41.3 (1.14)	43.0 (0.24)	ns	0.12	40.2 (1.04)	41.5 (0.25)	ns	0.11
Antisocial	43.5 (1.53)	46.3 (0.38)	ns	0.21	41.7 (1.44)	44.2 (0.37)	ns	0.20
Schizotypal	41.4 (1.70)	42.5 (0.33)	ns	0.08	39.6 (1.63)	41.0 (0.36)	ns	0.11

*Note:* All data weighted to adjust for nonresponse. Psychiatric categories not mutually exclusive. Lower scores indicate poorer perceived functioning. 2 < Cohen *d* < 5; Cohen *d* ≥ 5.

a Includes bulimia and anorexia nervosa.

The pattern was similar, also with smaller effect sizes, for the lifetime outcome variables: incarceration and violence to others (see Tables 3 and 4). Almost all forms of mental illness and alcohol abuse showed a small statistical effect in the direction of participants who described a legally significant outcome having greater impairment. The exception to this rule was role-emotional function and lifetime violence in schizophrenia/psychosis for which the effect size was moderate. There was no significant effect of social or role-emotional function in participants who did not have a mental disorder. Effect sizes were small or nonsignificant in drug abuse and the personality disorders.

**Table 3.** Lifetime incarceration (LI): effect size of functional impairment by lifetime diagnosis.

Lifetime diagnosis	Social functioning				Role-emotional functioning			
	LI present Mean (SE)	LI absent Mean (SE)	*p*	*d*	LI present Mean (SE)	LI absent Mean (SE)	*p*	*d*
No diagnosis	52.3 (0.38)	52.9 (0.09)	ns	0.07	50.2 (0.38)	50.3 (0.17)	ns	0.02
Any SU, MI, or PD	46.3 (0.29)	49.2 (0.15)	<0.0001	0.24	44.6 (0.29)	47.2 (0.14)	<0.0001	0.22
Any SU or MI	46.2 (0.29)	49.1 (0.15)	<0.0001	0.25	44.6 (0.29)	47.2 (0.14)	<0.0001	0.23
Any MI or PD	44.3 (0.34)	47.8 (0.16)	<0.0001	0.29	42.6 (0.31)	45.9 (0.14)	<0.0001	0.28
Any SU or PD	46.3 (0.29)	49.0 (0.16)	<0.0001	0.24	44.6 (0.30)	47.1 (0.15)	<0.0001	0.22
Comorbid SU and MI	43.0 (0.41)	46.6 (0.26)	<0.0001	0.29	41.5 (0.40)	45.1 (0.20)	<0.0001	0.31
Comorbid SU and PD	43.2 (0.45)	44.5 (0.33)	<0.05	0.10	41.9 (0.48)	43.1 (0.31)	<0.05	0.10
Comorbid MI and PD	41.0 (0.49)	43.2 (0.27)	<0.001	0.16	39.7 (0.48)	41.8 (0.23)	<0.001	0.17
Comorbid SU and PD and MI	41.3 (0.55)	42.9 (0.38)	<0.05	0.12	40.0 (0.58)	41.7 (0.33)	<0.05	0.14
Any MI	43.2 (0.37)	47.7 (0.17)	<0.0001	0.36	41.6 (0.34)	45.8 (0.15)	<0.0001	0.35
Schizophrenia/Psychosis	38.0 (1.30)	44.3 (0.86)	<0.0001	0.44	37.2 (1.20)	42.4 (0.96)	<0.001	0.39
Any mood disorder	42.0 (0.43)	47.0 (0.19)	<0.0001	0.40	40.5 (0.40)	45.1 (0.17)	<0.0001	0.39
Major depression	42.9 (0.51)	47.4 (0.17)	<0.0001	0.37	41.4 (0.47)	45.7 (0.16)	<0.0001	0.37
Persistent depressive	39.0 (0.92)	42.8 (0.41)	<0.001	0.27	36.2 (0.89)	41.5 (0.39)	<0.0001	0.41
Bipolar 1	38.7 (1.19)	44.1 (0.76)	<0.001	0.39	37.0 (1.31)	41.3 (0.62)	<0.01	0.34
Any anxiety disorder	41.2 (0.57)	46.5 (0.23)	<0.0001	0.41	40.0 (0.52)	44.7 (0.19)	<0.0001	0.38
Specific phobia	41.1 (1.01)	46.8 (0.36)	<0.0001	0.44	40.9 (0.89)	45.2 (0.33)	<0.0001	0.37
Social anxiety	37.4 (0.90)	43.9 (0.47)	<0.0001	0.48	37.6 (1.01)	43.2 (0.44)	<0.0001	0.44
Panic	37.1 (1.01)	43.5 (0.46)	<0.0001	0.46	36.6 (0.88)	42.3 (0.42)	<0.0001	0.45
Agoraphobia	35.5 (1.32)	41.9 (0.63)	<0.0001	0.46	34.8 (1.33)	40.1 0.66)	<0.001	0.41
Generalized anxiety	40.0 (0.83)	45.0 (0.32)	<0.0001	0.38	38.0 (0.74)	43.3 (0.25)	<0.0001	0.43
Posttraumatic stress	38.7 (0.86)	43.2 (0.38)	<0.0001	0.32	37.0 (0.74)	42.2 (0.32)	<0.0001	0.40
Eating disorder^a^	41.1 (1.58)	45.9 (0.67)	<0.01	0.37	40.1 (1.36)	45.0 (0.66)	<0.05	0.40
Any substance use disorder	46.7 (0.30)	49.7 (0.18)	<0.0001	0.27	45.1 (0.32)	47.8 (0.16)	<0.0001	0.25
Alcohol abuse	46.7 (0.30)	50.0 (0.18)	<0.0001	0.29	45.2 (0.33)	48.1 (0.17)	<0.0001	0.27
Drug abuse	45.2 (0.41)	46.9 (0.36)	<0.01	0.14	43.8 (0.48)	45.5 (0.29)	<0.01	0.14
Any personality disorder	43.2 (0.40)	45.0 (0.24)	<0.001	0.14	41.7 (0.40)	43.4 (0.22)	<0.001	0.13
Borderline	41.4 (0.47)	43.4 (0.28)	<0.001	0.16	40.0 (0.49)	42.1 (0.26)	<0.001	0.17
Antisocial	44.8 (0.59)	47.0 (0.49)	<0.01	0.17	43.2 (0.52)	44,5 (0.48)	ns	0.11
Schizotypal	39.5 (0.71)	43.6 (0.37)	<0.0001	0.30	38.6 (0.66)	41.8 (0.37)	<0.0001	0.24

*Note: All data weighted to adjust for nonresponse. Psychiatric categories not mutually exclusive. Lower scores indicate poorer perceived functioning. 2 < Cohen d < 5; Cohen d ≥ 5.*

a Includes bulimia and anorexia nervosa.

**Table 4.** Lifetime violence (LV) to others: effect size of functional impairment by lifetime diagnosis.

Lifetime diagnosis	Social functioning				Role-emotional functioning			
	LV present Mean (SE)	LV absent Mean (SE)	*p*	*d*	LV present Mean (SE)	LV absent Mean (SE)	*p*	*d*
No diagnosis	52.4 (0.30)	52.9 (0.10)	ns	0.05	50.0 (0.32)	50.4 (0.18)	ns	0.04
Any SU, MI, or PD	47.0 (0.18)	49.6 (0.15)	<0.0001	0.22	45.2 (0.20)	47.6 (0.15)	<0.0001	0.21
Any SU or MI	46.8 (0.19)	49.6 (0.15)	<0.0001	0.24	45.1 (0.20)	47.6 (0.14)	<0.0001	0.22
Any MI or PD	46.0 (0.19)	48.1 (0.18)	<0.0001	0.18	44.1 (0.20)	46.2 (0.17)	<0.0001	0.18
Any SU or PD	46.7 (0.20)	49.6 (0.17)	<0.0001	0.24	45.0 (0.21)	47.7 (0.16)	<0.0001	0.24
Comorbid SU and MI	44.6 (0.24)	46.9 (0.31)	<0.0001	0.18	42.9 (0.26)	45.4 (0.26)	<0.0001	0.22
Comorbid SU and PD	44.2 (0.26)	43.6 (0.54)	ns	0.05	42.7 (0.30)	42.7 (0.44)	ns	0.00
Comorbid MI and PD	42.6 (0.28)	42.5 (0.39)	ns	0.12	41.1 (0.26)	41.4 (0.33)	ns	0.03
Comorbid SU and PD and MI	42.5 (0.32)	42.0 (0.62)	ns	0.03	43.3 (0.22)	46.1 (0.17)	<0.0001	0.24
Any MI	45.1 (0.20)	48.0 (0.18)	<0.0001	0.24	43.3 (0.22)	46.1 (0.17)	<0.0001	0.24
Schizophrenia/Psychosis	39.2 (1.04)	45.6 (0.97)	<0.0001	0.45	37.2 (0.91)	44.3 (1.07)	<0.0001	0.54
Any mood disorder	44.3 (0.25)	47.3 (0.22)	<0.0001	0.24	42.7 (0.26)	45.5 (0.19)	<0.0001	0.24
Major depression	45.0 (0.28)	47.7 (0.21)	<0.0001	0.22	43.4 (0.26)	45.9 (0.18)	<0.0001	0.22
Persistent depressive	40.9 (0.54)	43.0 (0.50)	<0.01	0.15	39.7 (0.48)	41.3 (0.49)	<0.05	0.12
Bipolar 1	41.5 (0.72)	44.3 (1.22)	ns	0.20	39.4 (0.76)	41.3 (0.78)	ns	0.15
Any anxiety disorder	43.3 (0.31)	47.1 (0.25)	<0.0001	0.30	41.7 (0.32)	45.3 (0.22)	<0.0001	0.30
Specific phobia	43.5 (0.53)	47.4 (0.39)	<0.0001	0.31	42.2 (0.48)	46.1 (0.38)	<0.0001	0.33
Social anxiety	40.2 (0.69)	44.4 (0.57)	<0.0001	0.31	39.8 (0.63)	43.7 (0.59)	<0.0001	0.31
Panic	39.4 (0.58)	44.5 (0.52)	<0.0001	0.36	38.8 (0.64)	43.1 (0.44)	<0.0001	0.34
Agoraphobia	37.5 (0.91)	43.4 (0.86)	<0.0001	0.43	35.8 (0.76)	41.9 (0.79)	<0.0001	0.48
Generalized anxiety	42.1 (0.48)	45.7 (0.35)	<0.0001	0.28	40.2 (0.43)	44.0 (0.33)	<0.0001	0.30
Posttraumatic stress	40.9 (0.43)	43.7 (0.53)	<0.0001	0.21	39.8 (0.39)	42.5 (0.47)	<0.0001	0.21
Eating disorder^a^	42.9 (0.89)	46.7 (0.84)	<0.001	0.30	42.3 (0.94)	45.6 (0.77)	<0.01	0.27
Any substance use disorder	47.1 (0.20)	50.2 (0.17)	<0.0001	0.28	45.5 (0.23)	48.3 (0.17)	<0.0001	0.27
Alcohol abuse	47.4 (0.20)	50.4 (0.18)	<0.0001	0.28	45.7 (0.25)	48.5 (0.17)	<0.0001	0.27
Drug abuse	45.6 (0.32)	47.2 (0.36)	<0.001	0.13	44.0 (0.35)	45.9 (0.30)	<0.0001	0.17
Any personality disorder	44.6 (0.23)	44.3 (0.33)	ns	0.02	42.8 (0.24)	43.0 (0.30)	ns	0.00
Borderline	43.1 (0.26)	42.4 (0.44)	ns	0.05	41.5 (0.29)	41.4 (0.36)	ns	0.00
Antisocial	45.8 (0.40)	47.2 (1.02)	ns	0.11	43.7 (0.41)	45.5 (0.86)	ns	0.15
Schizotypal	41.5 (0.44)	44.0 (0.43)	<0.0001	0.18	40.0 (0.43)	42.4 (0.43)	<0.0001	0.18

*Note:* All data weighted to adjust for nonresponse. Psychiatric categories not mutually exclusive. Lower scores indicate poorer perceived functioning. 2 < Cohen *d* < 5; Cohen *d* ≥ 5.

a Includes bulimia and anorexia nervosa.

In light of previous suggestions of a curvilinear relationship between function and violence in psychosis we divided role-emotional function scores of participants with schizophrenia/psychosis into quintiles and calculated the prevalence of serious trouble with the police or the law in each quintile. The numbers were smaller than for other diagnostic categories (see Table 1). With that caveat, we did not find evidence of a curvilinear relationship (prevalence of serious trouble in five centiles, lowest functioning to highest, 32.1, 25, 17.9, 14.3, and 10.7%).

## Discussion

In a large and representative sample of the US population, people with mental disorders who report recent and serious trouble with the police or law, lifetime incarceration or lifetime violence to others also report impaired social and role-emotional functioning as consequence of emotional problems compared with people with mental disorders who do not report these outcomes. The association is statistically significant, diagnosis dependent and of up to moderate effect size. People without a mental disorder do not show it.

Because the NESARC-III obtained data at a single interview, participants’ functional impairment could have started before, during or after any of the three forms of legal involvement that we measured. This is particularly the case for the two lifetime measures (Tables 3 and 4). However, most of the diagnoses studied are either chronic or relapsing conditions that could be expected to be present for much of a participant’s life and participants were asked to limit their descriptions of functional impairment to instances in which the impairment was the result of emotional problems. Especially for the 12-month measures, it is more likely than not that any functional impairment bore a temporal relationship to the outcome variable.

The association between legally significant outcomes and functional impairment is greatest for mental illness and smallest for the personality disorders. For substance use the picture is mixed. In alcohol use disorder, but not in drug use, function is significantly associated with all three outcomes. It is possible that the behavioral nexus between the possession and distribution of illegal drugs, on the one hand, and violence and legal involvement, on the other, generates a ceiling effect, an association between diagnosis, criminal behavior and legal trouble that is already too substantial to be increased by any additional functional impairment secondary to substance abuse.

Function is most strongly associated with legally significant outcomes in those diagnoses where the odds of the outcome are lowest. The two largest associations between impaired function and 12-month trouble with the police or law, for instance, are for specific phobias and eating disorders, diagnoses with the lowest rates of legal trouble (see Table 1). In drug abuse and the personality disorders, legally significant outcomes are more frequent but the statistical effects of function are either small or statistically insignificant. The contribution of impaired psychosocial function may be analogous to the contribution of mentally abnormal homicide to homicide rates internationally. The contribution of mentally abnormal homicide appears to be relatively constant in terms of numbers and is therefore smaller in percentage terms in jurisdictions where other risk factors are more common and homicide rates are higher [23].

The association between function and legally significant outcomes is larger for the 12-month data (Table 2) than for the lifetime data (Tables 3 and 4). Most mental conditions vary in severity over their course. Even if social and role-emotional function was exerting a direct effect on the legal involvement, that effect could be expected to be less evident when function is measured after, perhaps long after, behavior. Some people who were functionally impaired when they acted violently, for instance, will have improved by the time their function was assessed, reducing the statistical association.

What do these findings suggest about the mechanisms linking mental disorder to legal trouble, incarceration and violence? First, the variation in the size of the association across diagnoses suggests that multiple mechanisms are involved. Second, studies of delusions in patients who have recently been discharged from the hospital [24] and studies of court-involved people who describe symptoms and signs of serious mental illness [25] both suggest that it is relatively unusual for symptoms and signs of illness to cause violence directly. Our data are more consistent with the conclusions suggested by these studies, that causal links are multiple and indirect, for instance operating through the damage that mental illness can cause to the family and social networks that would otherwise protect against incarceration, violence and other forms of legal involvement.

These data make some alternative explanations for the associations between function and legally significant outcomes less likely. It is possible, for instance, that the experience of having serious trouble with the police or law colored participants’ perception of their own levels of function. But had this been the case we would have expected the association between function and legally significant outcomes to be evident in nondisordered participants also, albeit to a lesser degree given that some of those participants will not have experienced the “emotional problems” that SF-12v12 items ask about. It is also possible that the change in function was a consequence, rather than a cause, of being in trouble with the police or law [26]. Again, however, had this been the case we would have expected reduced function, albeit perhaps to a lesser degree, in nondisordered participants.

These findings warrant several caveats. First, NESARC-III data derive exclusively from interviews with participants. We were not able to augment the data using collateral sources, for instance regarding incarceration and legal contacts. Second, the “schizophrenia/psychosis” variable was generated from a single NESARC item and not from the AUDADIS interview. While the approach we used was the same as that used by other studies using this sample [27], an interview-derived measure might have generated different results. Third, while the measures that generated our social function and role-emotional function variables have been extensively tested and widely used, we were not able to use collateral data to supplement and corroborate participants’ self-report. Fourth, and particularly for disadvantaged groups, engaging in a criminal act is not a necessary pre-requisite for legal involvement. People with mental disorders are also more likely than others to be apprehended when they do commit a criminal offense [28].

Most people who suffer from mental disorders have no history of behaving violently. But understanding the reasons for the exceptions to this rule remains of importance to patients, to potential victims (most often family members) and to mental health services. Future studies could advance the field by addressing the caveats we list here. The larger task, however, is to establish the nature of the links, described here as likely to be indirect and multiple, between social and role-emotional functioning and violence. Studies that can do that are likely to require detailed measures of legal involvement, robust measurement of causal factors and, ideally, longitudinal designs.

## Data Availability

Data are available at https://www.niaaa.nih.gov/research/nesarc-iii.
