# Psychosocial function, legal involvement and violence in mental disorder – CORRIGENDUM

**DOI:** 10.1192/j.eurpsy.2022.6

**Published:** 2022-01-24

**Authors:** Alec Buchanan, Kelly E. Moore, Brian Pittman, Sherry A. McKee

**Keywords:** Diagnosis, violent behavior, psychosocial function, incarceration, arrest, corrigendum

This article contains an error in the first row and third column of Table 1. The table as published gives this value as 254; it should in fact be 154. The correct version of the table is below.

**Table 1.** Description of sample: diagnosis and outcomes variables (n = 36,293)

Diagnosis			Serious legal trouble past 12 m		Lifetime incarceration		Lifetime violence to others	
	n 12m	n life	n (%)	**OR (95% CI)** ^c^	n (%)	**OR (95% CI)** ^c^	n (%)	**OR (95% CI)** ^c^
No diagnosis	22618	17775	154 (0.7%)	Ref	809 (4.6%)	Ref	1660 (9.4%)	Ref
Any SU, MI or PD	13675	18518	502 (3.7%)	6.1*** (5.1; 7.4)	3321 (18.0%)	4.7*** (4.2; 5.2)	6547 (35.4%)	4.9*** (4.5; 5.3)
Any SU or MI	12126	17755	467 (3.9%)	6.4*** (5.3; 7.7)	3181 (18%)	4.7*** (4.2; 5.2)	6117 (34.5%)	4.7*** (4.3; 5.1)
Any MI or PD	10797	13843	394 (3.6%)	6.2*** (5.2; 7.5)	2402 (17.4%)	4.5*** (4.0; 5.1)	5419 (39.2%)	5.8*** (5.3; 6.2)
Any SU or PD	9514	13219	462 (4.9%)	8.3*** (6.9; 10.0)	3034 (23.1%)	6.3*** (5.6; 7.1)	5681 (43.1%)	6.6*** (6.1; 7.2)
Comorbid SU and MI	2214	5470	171 (7.7%)	14.0*** (11.3; 17.4)	1428 (26.2%)	7.4*** (6.6; 8.4)	2743 (50.2%)	8.8*** (8.1; 9.7)
Comorbid SU and PD	2039	3455	205 (10.1%)	19.5*** (15.7; 24.2)	1287 (37.4%)	13.2*** (11.5; 15.2)	2522 (73.0%)	24.3*** (21.5; 27.5)
Comorbid MI and PD	3480	4198	207 (5.9%)	10.6*** (8.5; 13.3)	1186 (28.3%)	8.9*** (7.8; 10.3)	1665 (20.6%)	17.3*** (15.4; 19.4)
Comorbid SU and PD and MI	1323	2671	132 (10.0%)	19.0*** (14.6; 24.6)	963 (36.1%)	12.4*** (10.7; 14.5)	1944 (72.8%)	23.7*** (20.8; 26.9)
Any MI	8532	12296	286 (3.4%)	5.6*** (4.6; 6.9)	1938 (15.8%)	4.0*** (3.6; 4.5)	4411 (35.9%)	5.0*** (4.6; 5.4)
Schizophrenia/Psychosis	337	902	28 (8.3%)	17.5*** (10.6; 28.9)	218 (24.2%)	6.5*** (4.9; 8.6)	381 (42.2%)	6.9*** (5.3; 9.0)
Any Mood Disorder	4894	8639	198 (4.0%)	7.1*** (5.7; 8.9)	1382 (16.1%)	4.1*** (3.6; 4.6)	3252 (37.7%)	5.4*** (4.9; 5.8)
Major Depression	3961	7430	149 (3.8%)	6.6*** (5.1; 8.4)	1061 (14.4%)	3.5*** (3.1; 4.0)	2584 (34.8%)	4.7*** (4.3; 5.2)
Persistent Depressive	1185	2017	52 (4.4%)	7.6*** (5.4; 10.8)	371 (18.5%)	5.0*** (4.2; 5.9)	849 (42.3%)	6.7*** (5.8; 7.7)
Bipolar 1	565	752	37 (6.5%)	12.7*** (8.0; 20.1)	235 (31.5%)	9.8*** (7.6; 12.5)	491 (65.5%)	17.0*** (14.0; 20.7)
Any Anxiety Disorder	4700	5989	155 (3.3%)	5.5*** (4.3; 7.0)	1108 (16.9%)	4.3*** (3.8; 4.9)	2313 (38.7%)	5.6*** (5.1; 6.2)
Specific Phobia	2035	2279	51 (2.5%)	4.3*** (3.0; 6.2)	353 (15.5%)	3.9*** (3.2; 4.6)	853 (37.5%)	5.4*** (4.7; 6.2)
Social Anxiety	980	1255	37 (3.8%)	5.9*** (3.9; 8.9)	274 (22.0%)	5.9*** (4.9; 7.2)	552 (44.1%)	7.0*** (6.0; 8.3)
Panic	1103	1811	52 (4.7%)	7.0*** (4.7; 10.3)	353 (19.6%)	5.2*** (4.2; 6.3)	797 (44.1%)	6.9*** (6.1; 7.9)
Agoraphobia	549	690	22 (4.0%)	6.1*** (3.5; 10.6)	158 (23.1%)	6.3*** (4.8; 8.3)	342 (49.7%)	8.4*** (7.0; 10.1)
Generalized Anxiety	1908	2708	82 (4.3%)	7.2*** (5.2; 10.0)	505 (18.7%)	4.8*** (4.1; 5.6)	1170 (43.2%)	6.7*** (6.0; 7.6)
Posttraumatic Stress	1778	2337	87 (4.9%)	7.7*** (5.6; 10.6)	531 (22.8%)	6.3*** (5.4; 7.4)	1248 (53.4%)	9.8*** (8.7; 11.1)
Eating Disorder^a^	385	615	6 (1.6%)	2.3 (1.0; 5.6)	89 (14.5%)	3.8*** (2.7; 5.2)	251 (40.8%)	6.2*** (5.1; 7.5)
Any Substance Use Disorder	5808	10929	352 (6.1%)	10.5*** (8.6; 12.7)	2671 (24.6%)	6.7*** (6.0; 7.6)	4449 (40.8%)	6.1*** (5.5; 6.7)
Alcohol Abuse	5133	10000	306 (6.0%)	10.2*** (8.3; 12.5)	2388 (24.0%)	6.6*** (5.8; 7.4)	4032 (40.4%)	6.0*** (5.4; 6.6)
Drug Abuse	1487	3548	154 (10.4%)	19.9*** (15.5; 25.5)	1342 (38.1%)	13.1*** (11.3; 15.1)	1960 (55.4%)	11.2*** (9.9; 12.5)
Any personality disorder^b^	NA	5745	315 (5.5%)	10.6*** (8.3; 13.5)	1650 (28.8%)	9.1*** (8.0; 10.4)	3754 (65.3%)	17.3*** (15.6; 19.1)
Borderline^b^	NA	4300	250 (5.8%)	11.3*** (8.9; 14.3)	1240 (28.9%)	9.4*** (8.2; 10.7)	2911 (67.7%)	19.4*** (17.4; 21.7)
Antisocial^b^	NA	1600	132 (8.3%)	16.6*** (12.2; 22.6)	714 (44.8%)	18.5*** (15.7; 21.8)	1356 (84.8%)	49.8*** (41.8; 59.4)
Schizotypal^b^	NA	2438	148 (6.1%)	12.7*** (9.7; 16.6)	685 (28.2%)	8.8*** (7.4; 10.5)	1522 (62.4%)	15.0*** (13.1; 17.1)

Sample is limited to people with data on functional impairment. Psychiatric categories are not mutually exclusive. Lower scores indicate poorer perceived functioning.

*p<.05, **p<.01, ***p<.001.

a Includes bulimia, anorexia nervosa.

b Only lifetime personality disorder diagnoses are available.

c Data weighted to adjust for non-response.
