# Psychosocial function, legal involvement and violence in mental disorder – CORRIGENDUM

**DOI:** 10.1192/j.eurpsy.2022.2358

**Published:** 2023-01-12

**Authors:** Alec Buchanan, Kelly E. Moore, Brian Pittman, Sherry A. McKee

**Keywords:** Diagnosis, violent behavior, psychosocial function, incarceration, arrest, corrigendum

This article was published with a small error in Table 1. The correct version of the table is below, with the corrected value highlighted.

**Table 1.** Description of sample: diagnosis and outcomes variables (n = 36,293)

Diagnosis			Serious legal trouble past 12 m		Lifetime incarceration		Lifetime violence to others	
	n 12m	n life	n (%)	OR (95% CI)^c^	n (%)	OR (95% CI)^c^	n (%)	OR (95% CI)^c^
No diagnosis	22618	17775	154 (0.7%)	Ref	809 (4.6%)	Ref	1660 (9.4%)	Ref
Any SU, MI or PD	13675	18518	502 (3.7%)	6.1*** (5.1; 7.4)	3321 (18.0%)	4.7*** (4.2; 5.2)	6547 (35.4%)	4.9*** (4.5; 5.3)
Any SU or MI	12126	17755	467 (3.9%)	6.4*** (5.3; 7.7)	3181 (18%)	4.7*** (4.2; 5.2)	6117 (34.5%)	4.7*** (4.3; 5.1)
Any MI or PD	10797	13843	394 (3.6%)	6.2*** (5.2; 7.5)	2402 (17.4%)	4.5*** (4.0; 5.1)	5419 (39.2%)	5.8*** (5.3; 6.2)
Any SU or PD	9514	13219	462 (4.9%)	8.3*** (6.9; 10.0)	3034 (23.1%)	6.3*** (5.6; 7.1)	5681 (43.1%)	6.6*** (6.1; 7.2)
Comorbid SU and MI	2214	5470	171 (7.7%)	14.0*** (11.3; 17.4)	1428 (26.2%)	7.4*** (6.6; 8.4)	2743 (50.2%)	8.8*** (8.1; 9.7)
Comorbid SU and PD	2039	3455	205 (10.1%)	19.5*** (15.7; 24.2)	1287 (37.4%)	13.2*** (11.5; 15.2)	2522 (73.0%)	24.3*** (21.5; 27.5)
Comorbid MI and PD	3480	4198	207 (5.9%)	10.6*** (8.5; 13.3)	1186 (28.3%)	8.9*** (7.8; 10.3)	2746 (65.4%)	17.3*** (15.4; 19.4)
Comorbid SU and PD and MI	1323	2671	132 (10.0%)	19.0*** (14.6; 24.6)	963 (36.1%)	12.4*** (10.7; 14.5)	1944 (72.8%)	23.7*** (20.8; 26.9)
Any MI	8532	12296	286 (3.4%)	5.6*** (4.6; 6.9)	1938 (15.8%)	4.0*** (3.6; 4.5)	4411 (35.9%)	5.0*** (4.6; 5.4)
Schizophrenia/Psychosis	337	902	28 (8.3%)	17.5*** (10.6; 28.9)	218 (24.2%)	6.5*** (4.9; 8.6)	381 (42.2%)	6.9*** (5.3; 9.0)
Any Mood Disorder	4894	8639	198 (4.0%)	7.1*** (5.7; 8.9)	1382 (16.1%)	4.1*** (3.6; 4.6)	3252 (37.7%)	5.4*** (4.9; 5.8)
Major Depression	3961	7430	149 (3.8%)	6.6*** (5.1; 8.4)	1061 (14.4%)	3.5*** (3.1; 4.0)	2584 (34.8%)	4.7*** (4.3; 5.2)
Persistent Depressive	1185	2017	52 (4.4%)	7.6*** (5.4; 10.8)	371 (18.5%)	5.0*** (4.2; 5.9)	849 (42.3%)	6.7*** (5.8; 7.7)
Bipolar 1	565	752	37 (6.5%)	12.7*** (8.0; 20.1)	235 (31.5%)	9.8*** (7.6; 12.5)	491 (65.5%)	17.0*** (14.0; 20.7)
Any Anxiety Disorder	4700	5989	155 (3.3%)	5.5*** (4.3; 7.0)	1108 (16.9%)	4.3*** (3.8; 4.9)	2313 (38.7%)	5.6*** (5.1; 6.2)
Specific Phobia	2035	2279	51 (2.5%)	4.3*** (3.0; 6.2)	353 (15.5%)	3.9*** (3.2; 4.6)	853 (37.5%)	5.4*** (4.7; 6.2)
Social Anxiety	980	1255	37 (3.8%)	5.9*** (3.9; 8.9)	274 (22.0%)	5.9*** (4.9; 7.2)	552 (44.1%)	7.0*** (6.0; 8.3)
Panic	1103	1811	52 (4.7%)	7.0*** (4.7; 10.3)	353 (19.6%)	5.2*** (4.2; 6.3)	797 (44.1%)	6.9*** (6.1; 7.9)
Agoraphobia	549	690	22 (4.0%)	6.1*** (3.5; 10.6)	158 (23.1%)	6.3*** (4.8; 8.3)	342 (49.7%)	8.4*** (7.0; 10.1)
Generalized Anxiety	1908	2708	82 (4.3%)	7.2*** (5.2; 10.0)	505 (18.7%)	4.8*** (4.1; 5.6)	1170 (43.2%)	6.7*** (6.0; 7.6)
Posttraumatic Stress	1778	2337	87 (4.9%)	7.7*** (5.6; 10.6)	531 (22.8%)	6.3*** (5.4; 7.4)	1248 (53.4%)	9.8*** (8.7; 11.1)
Eating Disorder^a^	385	615	6 (1.6%)	2.3 (1.0; 5.6)	89 (14.5%)	3.8*** (2.7; 5.2)	251 (40.8%)	6.2*** (5.1; 7.5)
Any Substance Use Disorder	5808	10929	352 (6.1%)	10.5*** (8.6; 12.7)	2671 (24.6%)	6.7*** (6.0; 7.6)	4449 (40.8%)	6.1*** (5.5; 6.7)
Alcohol Abuse	5133	10000	306 (6.0%)	10.2*** (8.3; 12.5)	2388 (24.0%)	6.6*** (5.8; 7.4)	4032 (40.4%)	6.0*** (5.4; 6.6)
Drug Abuse	1487	3548	154 (10.4%)	19.9*** (15.5; 25.5)	1342 (38.1%)	13.1*** (11.3; 15.1)	1960 (55.4%)	11.2*** (9.9; 12.5)
Any personality disorder^b^	NA	5745	315 (5.5%)	10.6*** (8.3; 13.5)	1650 (28.8%)	9.1*** (8.0; 10.4)	3754 (65.3%)	17.3*** (15.6; 19.1)
Borderline^b^	NA	4300	250 (5.8%)	11.3*** (8.9; 14.3)	1240 (28.9%)	9.4*** (8.2; 10.7)	2911 (67.7%)	19.4*** (17.4; 21.7)
Antisocial^b^	NA	1600	132 (8.3%)	16.6*** (12.2; 22.6)	714 (44.8%)	18.5*** (15.7; 21.8)	1356 (84.8%)	49.8*** (41.8; 59.4)
Schizotypal^b^	NA	2438	148 (6.1%)	12.7*** (9.7; 16.6)	685 (28.2%)	8.8*** (7.4; 10.5)	1522 (62.4%)	15.0*** (13.1; 17.1)

Sample is limited to people with data on functional impairment. Psychiatric categories are not mutually exclusive. Lower scores indicate poorer perceived functioning.

* p<.05

** p<.01

*** p<.001.

a Includes bulimia, anorexia nervosa.

b Only lifetime personality disorder diagnoses are available.

c Data weighted to adjust for non-response.
